# Pathological complete response of initially unresectable multiple liver metastases achieved using combined peptide receptor radionuclide therapy and somatostatin analogs following pancreatic neuroendocrine tumor resection: a case report

**DOI:** 10.1186/s40792-024-01839-4

**Published:** 2024-02-14

**Authors:** Ryosuke Umino, Satoshi Nara, Noritoshi Kobayashi, Takahiro Mizui, Takeshi Takamoto, Daisuke Ban, Minoru Esaki, Nobuyoshi Hiraoka, Kazuaki Shimada

**Affiliations:** 1 Department of Hepatobiliary and Pancreatic Surgery, National Cancer Center Hospital, 5-1-1, Tsukiji, Chuo-Ku, Tokyo, 104-0045 Japan; 2 Department of Oncology, Yokohama City University Graduate School of Medicine, Yokohama, Japan; 3 Department of Molecular Pathology, National Cancer Center Hospital, Tokyo, Japan

**Keywords:** PRRT, Pancreas, Neuroendocrine tumor, Liver metastasis, Conversion surgery, Pathological complete response, pCR

## Abstract

**Background:**

Peptide receptor radionuclide therapy (PRRT) serves as a novel and effective treatment option for somatostatin receptor-positive unresectable liver metastases of pancreatic neuroendocrine tumors (PNETs). However, there are few reported cases of surgical resection for initially unresectable liver metastases of PNET that were converted to resectable after PRRT. Here we report a case where PRRT and somatostatin analogs (SSAs) led to a pathological complete response of initially unresectable multiple liver metastases following PNET resection.

**Case presentation:**

A 52-year-old man underwent pylorus-preserving pancreaticoduodenectomy for PNET at age 40 and subsequent hepatectomies for resectable liver metastases at 44 and 47 years of age. At age 48, a follow-up examination revealed unresectable multiple liver metastases, and PRRT with ^177^Lu-DOTATATE therapy was initiated. After four cycles of PRRT, most liver metastases diminished according to imaging studies, and the remaining two hepatic lesions continued to shrink with additional lanreotide. Conversion surgery for liver metastases was successfully performed, revealing no viable tumor cells in tissue specimens. Seventeen months after surgery, imaging showed no detectable residual tumor or recurrence. We present a review of the relevant literature that highlights the significance of our findings.

**Conclusions:**

This rare case highlights the pathological complete response of initially unresectable multiple liver metastases achieved by PRRT and SSAs following PNET resection, suggesting their potential as a multimodality treatment option for unresectable PNET.

**Supplementary Information:**

The online version contains supplementary material available at 10.1186/s40792-024-01839-4.

## Introduction

Pancreatic neuroendocrine tumors (PNETs) are rare, accounting for approximately 1–2% of pancreatic tumors [1]. Patients diagnosed with PNET experience 5-year survival rates ranging from 23% to 95% [2] that are significantly longer than those for pancreatic ductal adenocarcinoma. Despite the relatively indolent clinical courses of the disease, PNETs frequently manifest with distant metastases, with liver metastases the most common, affecting approximately 30%–85% of patients who are faced with a grim prognosis [3, 4]. Liver resection achieves significant survival benefits by reducing tumor burden and slowing disease progression, resulting in favorable 5-year survival rates ranging from 60% to 80% [3, 5]. Consequently, global practice recommends liver resection for such patients; however, for most cases, liver resection is often considered inappropriate, particularly in the presence of extrahepatic lesions and a high tumor burden across both liver lobes [6, 7]. In such cases, the cornerstone of treatment involves multimodal therapy, including systemic chemotherapy, transcatheter arterial chemoembolization, and ablation.

Peptide receptor radionuclide therapy (PRRT) selectively attacks tumor cells by binding to peptides expressed on their surface [8]. NETs frequently express membrane-localized somatostatin receptors (SSTRs), making these receptors suitable targets for PRRT using somatostatin analogs (SSAs). PRRT has been predominantly employed in Europe since the late 1990s to stabilize unresectable advanced or end-stage NETs. Previous studies report high response rates and favorable long-term outcomes with PRRT in patients with NET [9–12]. Consequently, PRRT is unequivocally recognized in multiple guidelines as a novel and effective treatment option for SSTR-positive and progressive NETs [13–16]. A similar favorable morphological response occurs in liver metastases of PNET, suggesting potential improvement in overall survival (OS) and progression-free survival (PFS) [17, 18]. However, the cases [19–21] in which initially unresectable liver metastases of PNET are converted to resectable after PRRT, followed by successful resection (conversion surgery), are limited given the high tumor burden and remnant liver function.

Here we report a case of conversion surgery for a patient who developed multiple unresectable liver metastases after PNET resection and underwent liver resection after PRRT with an SSA, leading to a remarkable reduction of tumor burden. We describe the pathological findings for the resected liver specimens and present a review of the relevant literature regarding patients who underwent conversion surgery after PRRT.

## Case report

### Initial diagnosis and pancreaticoduodenectomy

**Fig. 1:**
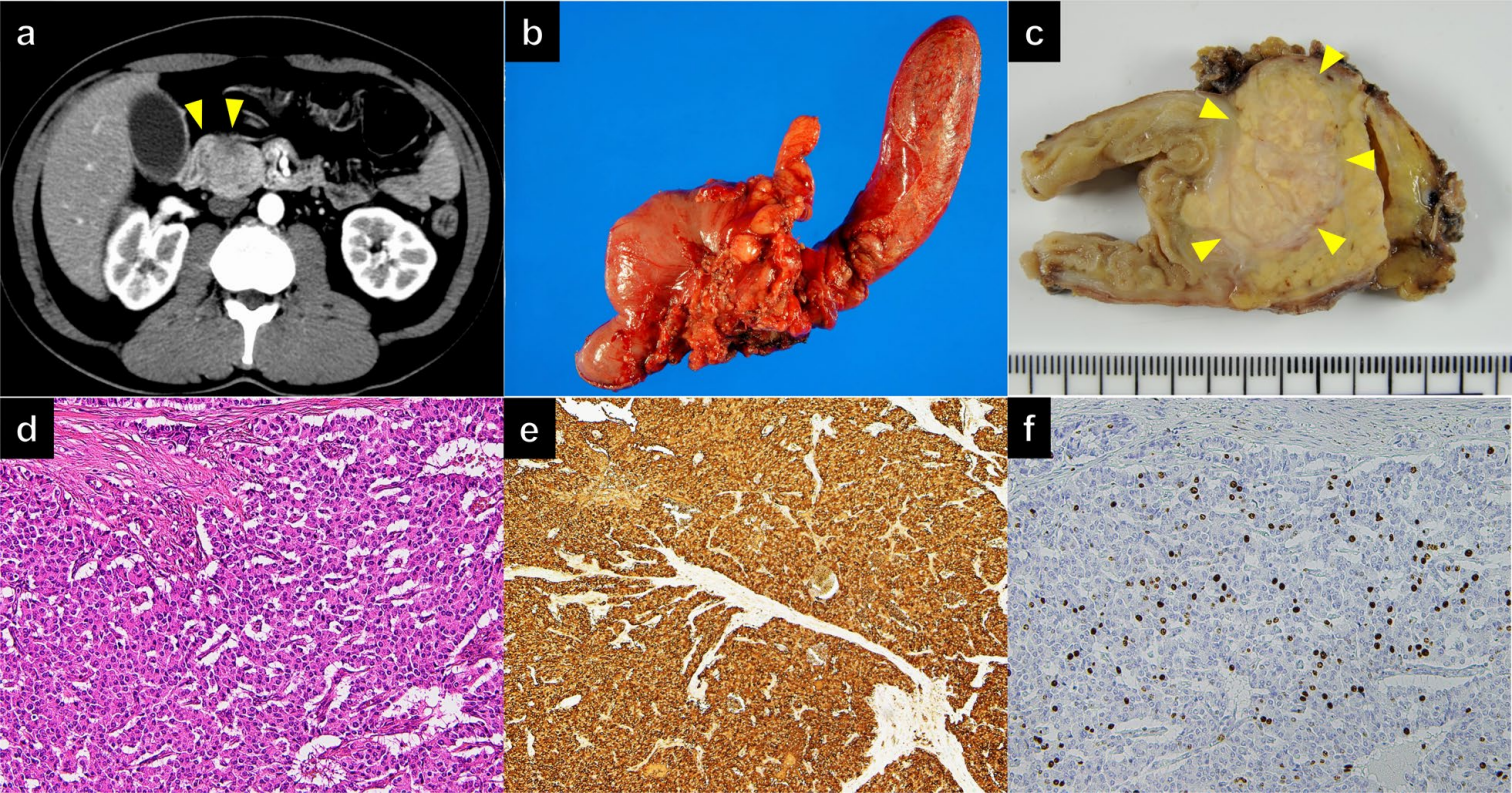
Primary pancreatic neuroendocrine tumor. **a** Contrast-enhanced computed tomography demonstrated a hypo-attenuated mass in the pancreatic head (arrowheads). **b** Gross appearance of the specimen obtained by pancreatoduodenectomy. **c** On the cut section, a whitish-yellow nodular tumor (arrowheads) appeared in the pancreatic parenchyma and invaded the duodenal wall. **d–f** Histology (**d**) and immunohistochemistry (**e, f**) of the resected specimen in medium-power views. Atypical polygonal proliferating tumor cells arranged in trabecular or nested patterns (hematoxylin and eosin staining) (**d**). Most tumor cells expressed chromogranin A (**e**), and their Ki67 labeling index was approximately 10% (**f**)

A 40-year-old male with jaundice was referred to our hospital for further evaluation. During the initial visit, contrast-enhanced computed tomography (CECT) demonstrated a 35-mm hypo-attenuated mass in the pancreatic head (Fig. 1a). No obvious regional lymph node metastasis or distant metastasis was detected. Although there was no histological confirmation due to the absence of a tumor biopsy, the radiological findings strongly indicated the presence of invasive pancreatic ductal adenocarcinoma. Subsequently, the patient underwent pylorus-preserving pancreaticoduodenectomy (PPPD) with lymph node dissection, which achieved R0 resection (Fig. 1b). Histopathological diagnosis revealed that the tumor was a PNET G2 (3.5 cm; mitotic count 6 per 10 high-power fields; Ki67 proliferative index, 10%) (Fig. 1c–f). According to the UICC TNM classification of malignant tumors 8th edition [22], the PNET was classified as pT3N0M0 Stage II with invasion into the common bile duct’s epithelium and the duodenum’s submucosa.

### Resectable liver metastasis and hepatectomy

**Fig. 2:**
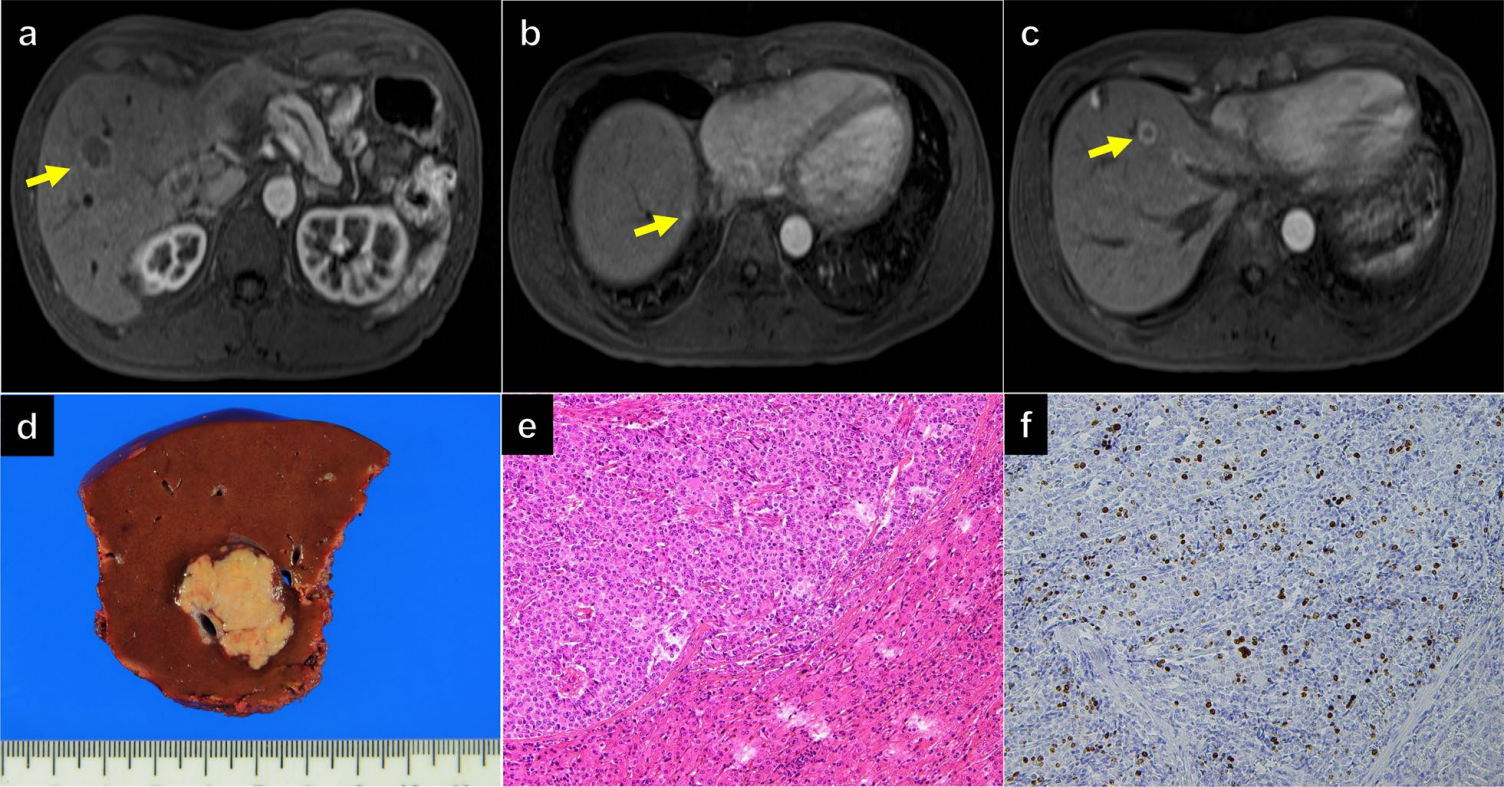
Initial recurrence. **a–c** Magnetic resonance imaging revealed multiple liver metastases (arrow). **d** A fresh cut specimen of the well-demarcated whitish-yellow nodular tumor in liver segment 5, showing a solid tumor without necrosis. **e, f** Histology (**e**) and immunohistochemistry of Ki67 (**f**) of the resected specimen in medium power views. Histological features of proliferating tumor cells arranged in trabecular and nested patterns were similar to those of the primary tumor (**e**). Ki-67 labeling index of the metastatic liver tumor was 12% (**f**)

**Fig. 3:**
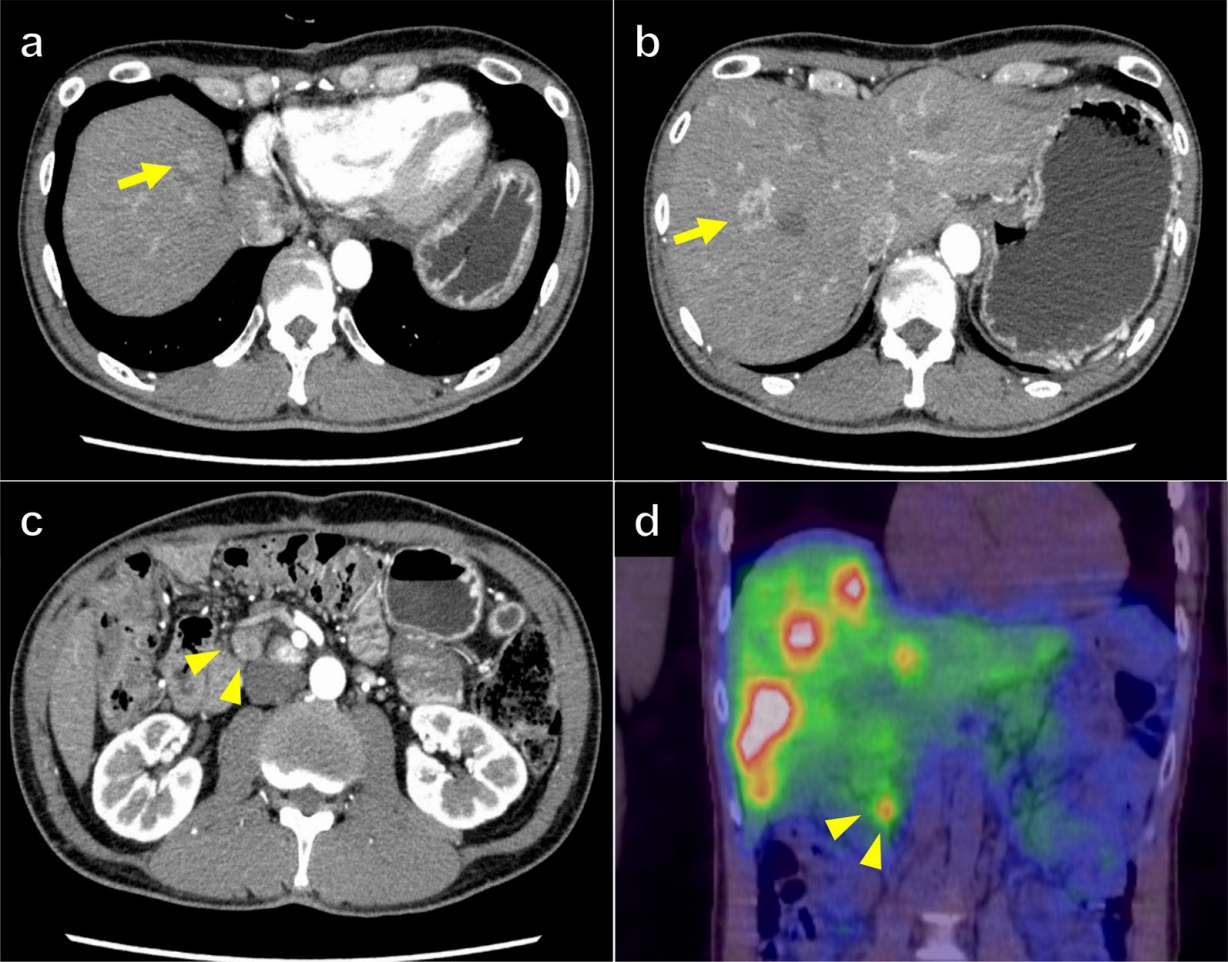
Second recurrence. Contrast-enhanced computed tomography revealed multiple liver metastases (arrows) (**a** and **b**) and lymph node metastasis near the superior mesenteric vein (arrowheads) (**c**). Somatostatin receptor scintigraphy revealed receptor accumulation in the liver and the lymph node (arrowheads) (**d**)

The patient underwent regular postoperative follow-up imaging examinations. Six liver lesions were detected in segments (S) 5, 7, and 8 (Fig. 2a–c) after 45 months, raising suspicion of multiple metastases of PNET. Evaluating the resectability of the lesions and considering the patient’s liver function assessed through indocyanine green clearance (R15: 3.3%, K: 0.22709, Child–Pugh classification A), we performed three partial hepatectomies. Histopathological analysis confirmed the diagnosis of PNET metastases for all lesions, classified as G2 (Ki67 index, 12%) (Fig. 2d–f). Approximately one year after liver resection, magnetic resonance imaging (MRI) revealed multiple liver metastases and enlarged mesenteric lymph nodes. Due to the small size of the liver metastases, observation was planned without immediate resection. After careful monitoring, the lesions exhibited slight growth over 17 months, making them amenable to resection (Fig. 3a–c). Somatostatin receptor scintigraphy (SRS) with ^111^In-pentetreotide and single photon emission computed tomography (SPECT) showed multiple liver metastases with substantial SRS detection of accumulation (scores 3–4) in the early phase [23]. Furthermore, in the delayed phase, SRS detected accumulation in the lymph node near the superior mesenteric vein as well as in the liver lesions (Fig. 3d). Subsequently, 13 partial liver resections and mesenteric lymphadenectomies were performed, yielding 18 liver metastases and one mesenteric lymph node metastasis.

### PRRT and SSAs for unresectable liver metastases

**Fig. 4:**
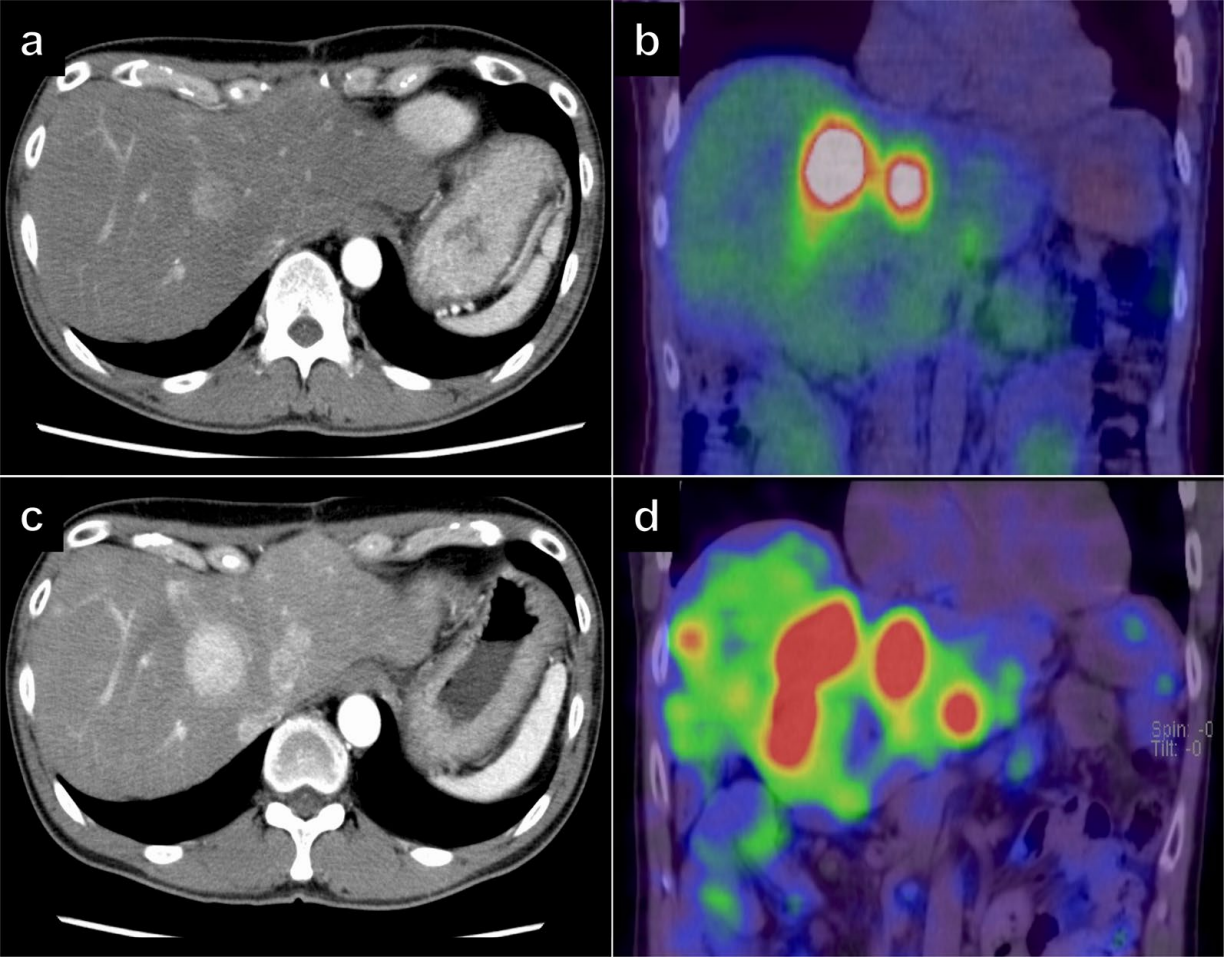
Third recurrence. Contrast-enhanced computed tomography revealed multiple liver metastases (**a**), and somatostatin receptor scintigraphy confirmed the abnormal accumulation in the tumor (**b**). Despite administering lanreotide, the tumor progressed (**c** and **d**)

**Fig. 5:**
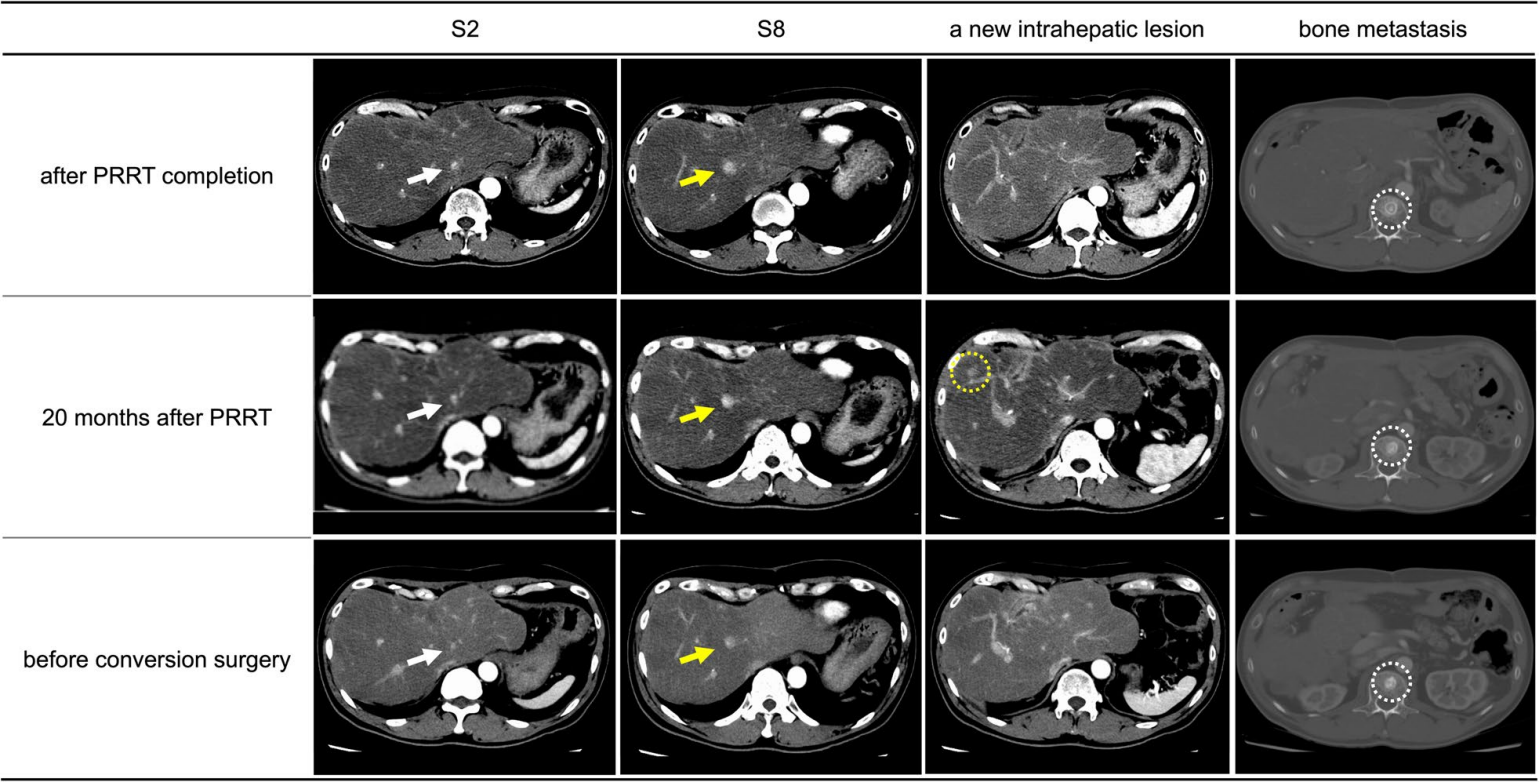
Post-PRRT to conversion surgery (contrast-enhanced computed tomography images). Most liver metastases diminished on imaging during peptide receptor radionuclide therapy, and only two reduced lesions with blurred contrast enhancement were detected in segments 2 (white arrow) and 8 (yellow arrow). A new intrahepatic lesion was identified 20 months after completion of PRRT (yellow dotted circle), however it disappeared after lanreotide administration. The bone metastasis remained stable during the treatment (white dotted circle)

**Fig. 6:**
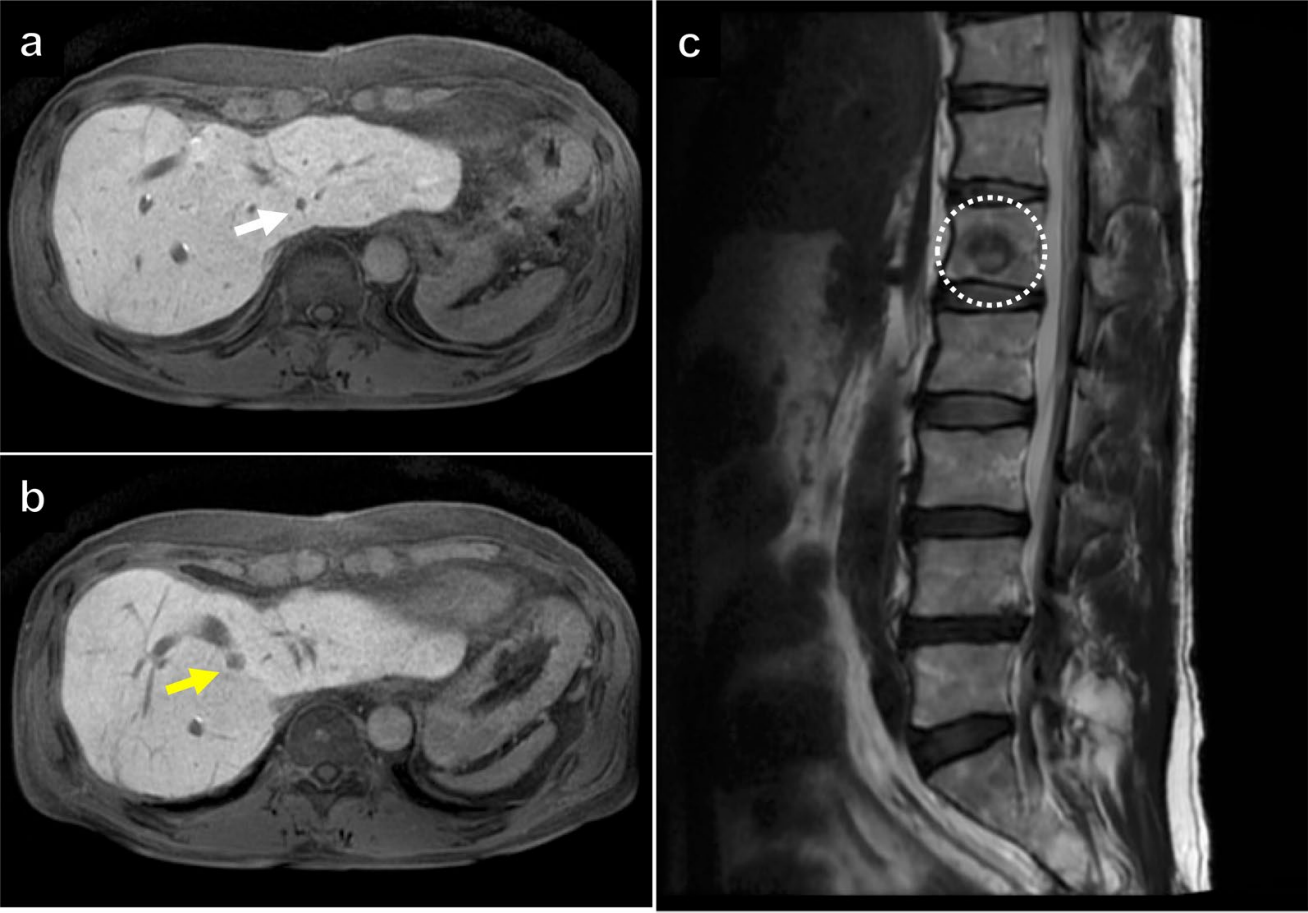
Gadolinium-ethoxybenzyl-diethylene-triamine-pentaacetic acid enhanced magnetic resonance imaging revealed only two liver metastases in segment 2 (**a** white arrow) and 8 (**b** yellow arrow). The bone metastasis had no remarkable change in size (**c** white dotted circle)

Eleven months after the second hepatectomy, CT revealed 16 multiple liver metastases and bone metastasis in the first lumbar vertebra (L1). SRS detected multiple nodules with score 3 as well as the accumulation in the liver and L1 vertebral body, indicating liver and bone metastases (Fig. 4a, b). The liver metastases were judged unresectable due to the presence of extrahepatic lesions. The patient was administered the SSA lanreotide, but the sizes of the lesions increased (Fig. 4c, d). Subsequently, the patient expressed interest in PRRT and was referred to another hospital. To initiate PRRT therapy, the patient provided informed consent to participate in the F-1515 phase I/II clinical trial [24]. The patient had no adverse events during PRRT. Following the administration of four cycles of PRRT with ^177^Lu-DOTATATE, most liver metastases diminished on imaging, and the remaining two hepatic lesions continued to shrink. While the liver metastases remained under control for a prolonged period, a new intrahepatic lesion was suspected 20 months after the completion of PRRT. After reintroducing lanreotide, the new intrahepatic lesion disappeared, and we could visualize only previously detected two lesions. Bone metastasis was well controlled with no remarkable change (Fig. 5). In the referral hospital, continued lanreotide therapy was proposed because there was no long-term progression of both liver and bone metastases. However, the patient wished to undergo tumor resection if possible, and further evaluation was performed in the referral hospital. A gadolinium-ethoxybenzyl-diethylene-triamine-pentaacetic acid-enhanced MRI showed no apparent liver metastasis other than the two lesions. In addition, MRI showed no change in the size of bone metastasis and no other extrahepatic lesions except for the bone metastasis (Fig. 6). Based on these findings, the disease was considered stable, and we thought the surgical resection of the remaining liver metastases was appropriate.

### Conversion surgery for liver metastases

**Fig. 7:**
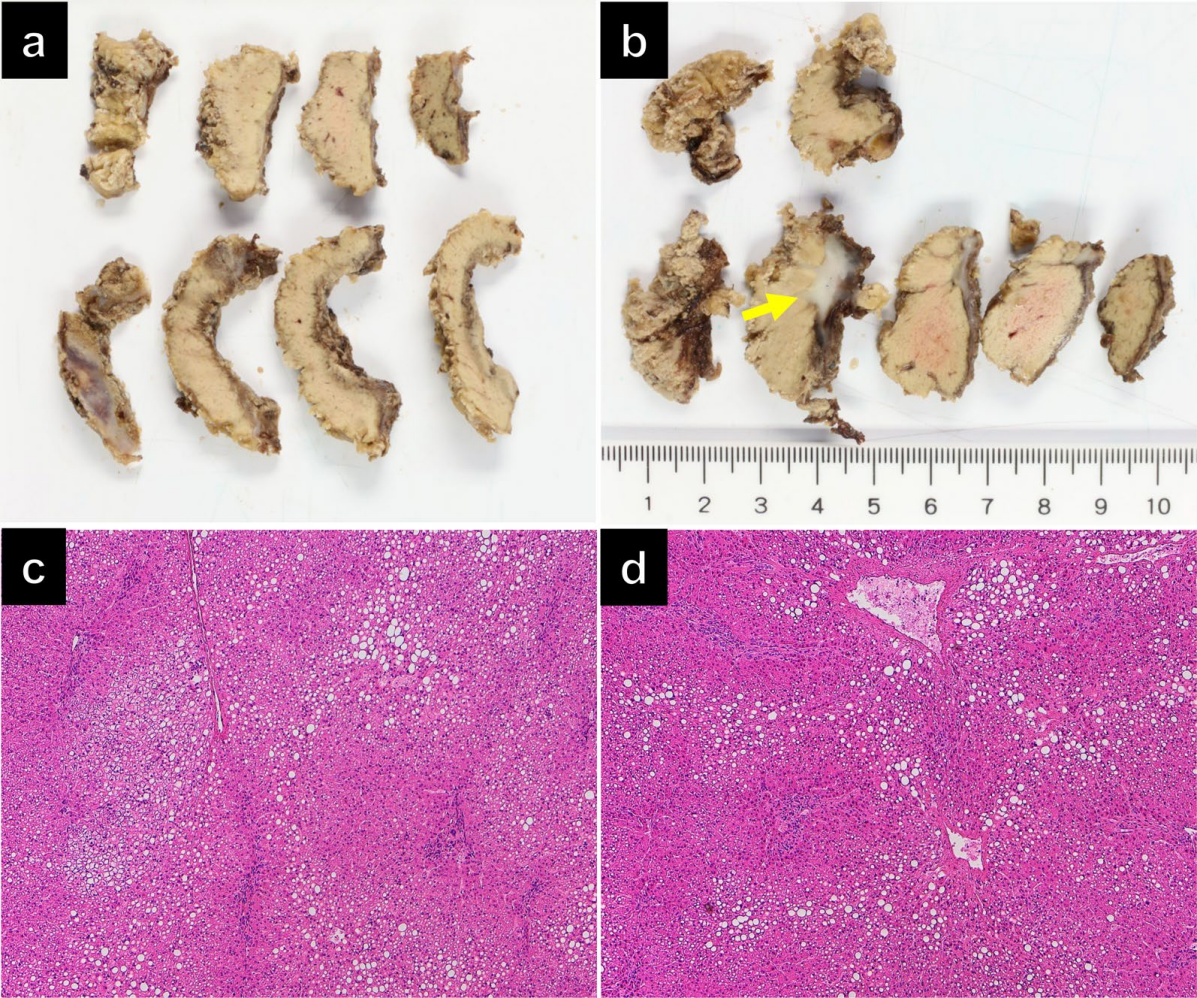
Cut surface of formalin-fixed specimens (**a, b**). The specimens resected by partial hepatectomies of segment 2 (**a**) and segment 8 (**b**) were cut into serial slices 5-mm thick. All sections underwent histological examination after staining with hematoxylin and eosin (**c, d**). No viable or degenerated/necrotic tumor cells were found. A whitish fibrotic nodule in a section (arrow) of segment 8 was a histologically fibrotic scar (**b**)

After being referred again to our hospital, the patient underwent a partial hepatectomy (S2 and S8). This marked the third liver resection, and severe intraperitoneal adhesions were noted during the procedure. Intraoperative ultrasound findings indicated the absence of tumors, except for the two lesions, which were difficult to distinguish between scars or tumors. Therefore, two partial hepatectomies were performed. Because the tumors were difficult to detect in the resected specimens from both S2 and S8, additional liver resections were conducted to fully include the tumor area respectively. Operative time was 557 min, and blood loss was 1892 mL. There were no postoperative complications, and the patient was discharged on postoperative day 10. The resected specimens were S2 (2.5 × 1.5 × 1.5 cm, 5.0 × 2.5 × 0.5 cm) and S8 (3.5 × 3.0 × 1.0 cm, 3.0 × 2.5 × 0.6 cm). Gross examination revealed the presence of a grayish-white lesion resembling scar tissue on the surface of the liver S8 specimen; however, no apparent tumor was identified (Fig. 7a, b). Histopathological analysis did not detect viable tumor cells in both specimens (Fig. 7c, d). Seventeen months after surgery, the bone metastasis remained stable, and no other recurrence was identified via imaging modalities, including CECT and SRS, without administering drug therapy. The patient’s clinical course is summarized in Additional file 1: Figure S1.

## Discussion

We present here the case of a patient who underwent PRRT for multiple unresectable liver metastatic recurrences after PNET resection. Following PRRT and SSA treatment, 16 liver metastases exhibited marked reduction, and partial hepatectomy was performed on the remaining two lesions. Notably, pathological examination of the resected liver specimens revealed no residual viable tumor cells. This case is noteworthy for the reasons as follows. First, it is uncommon for unresectable lesions, whether locally advanced or distant metastatic NET, to be resected after a favorable response to PRRT, i.e. conversion surgery. Second, the histological diagnosis of pathological complete response (pCR) makes this case highly rare; and to our knowledge, no reports exist regarding pCR for initially unresectable liver metastases in patients with PNETs. Third, to date, no tumor regrowth or new metastatic lesions have been identified 17 months after the final surgery.

As a consequence of recent advances in enhancing the efficacies of chemotherapy and radiotherapy, an increasing number of conversion surgeries have been applied to patients with colorectal liver metastases, pancreatic cancer, and gastric cancer, thus becoming a common treatment strategy [25, 26]. In PNETs, conversion surgery following chemotherapy, such as with sunitinib, streptozocin, or capecitabine/temozolomide, offer an improved prognosis [27, 28]. PRRT with ^90^Y- or ^177^Lu-DOTATATE achieve better response rates and longer PFS for patients with unresectable NETs than the aforementioned chemotherapies [11, 29, 30]. Thus, conversion of unresectable to resectable disease after PRRT is occasionally observed for some patients [19–21, 31–33].

A literature search for conversion surgery after PRRT revealed 9 cases, including that of our patient [19–21, 31–33]. Furthermore, Staszczak et al. presented a case in which tumor size reduction enabled surgical intervention [34]. However, surgery had not been performed at the time of the report and was under follow-up. As shown in Table 1, six of the nine patients showed a CR or partial response after PRRT for primary and distant metastatic disease, and one patient was diagnosed with progressive disease due to the emergence of a new liver lesion. Seven patients achieved R0 resection, and none experienced tumor recurrence during follow-up. Including our case, there were six patients with liver metastases; five patients who underwent liver resection, and four patients who achieved R0 resection. In one patient without R0 liver resection, three residual tumors continued to exhibit excellent responses after surgery. However, tumor regrowth was observed after 57 months. Subsequently, PRRT was repeated, and all tumors regressed. One patient did not undergo liver resection because a radiological CR was obtained after PRRT. There was no evidence of liver metastasis 22 months after surgery. Our case confirmed a pCR was achieved by PRRT and SSA treatment, contrary to other cases that show residual tumor cells in the resected specimen [19–21]. In summary, multiple patients with unresectable liver metastases of PNETs achieved significant responses to PRRT, highlighting the crucial role of PRRT as a multimodal treatment option in PNETs with liver metastases. Furthermore, it is noteworthy that conversion surgery can provide the advantage of liberation from long-term drug treatment, as demonstrated here.

**Table 1:** Conversion surgery after PRRT for initially unresectable PNET

Case	Year	Authors	Age	Sex	Liver metastasis	Unresectable factors	Pre-treatment	Response (RECIST)	Conversion surgery	R	PFS months
1	2009	Kaemmerer [31]	33	F	No	SMA/SMV involvement PALN metastasis	^90^Y-DOTATATE × 2cycles	PR	PPPD + Mesenterium LN resection	R0	18.0
2	2010	Stoeltzing [19]	49	M	Yes	Multiple liver metastases	Distal pancreatectomy → ^90^Y-DOTATOC × 2cycles	PR	Subsegmentectomy + RFA	R0	12.0
3	2012	Ezziddin [32]	43	F	Yes	SMV involvement Multiple liver metastases	^177^Lu-DOTATATE × 3cycles	PR-CR	PD + adjuvant SSA	R0	22.0
4	2012	Barber [33]	50	M	No	Not mentioned	Chemotherapy → ^177^Lu-DOTATATE × 4cycles	PR	Not mentioned	R0	12.0
5	2020	Chiapponi [20]	49	M	Yes	Multiple liver metastases	Sandostatin + ^177^Lu-DOTATATE → Selective internal radiotherapy	PR	Distal pancreatectomy + Partial hepatectomy	R1	57.0^*^
6	2021	Opalinska [21]	–	M	No	Large vessel infiltration or Invasion to adjacent organs	^90^Y-DOTATATE^¶^	PD	Not mentioned	R1	8.2
7	2021	Opalinska [21]	–	M	Yes		^177^Lu/^90^Y-DOTATATE^¶^	SD	Hemi-hepatectomy	R0	116.2
8	2021	Opalinska^21^	–	F	Yes		^90^Y-DOTATATE^¶^	SD	Not mentioned	R0	26.4
9	2023	Our case	52	M	Yes	Bone metastasis Multiple liver metastases	SSA → ^177^Lu-DOTATATE + SSA	PR-CR	Partial hepatectomy	R0	17.0
10	2011	Staszczak^34^	56	M	Yes	SMV involvement Multiple liver metastases	^90^Y-DOTATATE^¶^	PR	Not performed	–	–

*RECIST* Response Evaluation Criteria In Solid Tumors, *CR *complete response, *PR* Partial response, *SD* stable disease, *PD* progression disease, *PFS *progression-free survival, *SMA* superior mesenteric artery, *SMV *superior mesenteric vein, *SSA* somatostatin analog, *PPPD* pyrolus-preserving pancreaticoduodenectomy, *PALN* para aortice lymph node, *RFA* radiofrequency ablation

^¶^Patients may have received chemotherapy or somatostatin analogs before PRRT

*All the tumors had regressed after PRRT was performed again

However, it is essential to recognize that the effectiveness of PRRT is not uniform among all NETs [9, 10, 17] due to their diverse and heterogeneous nature. Moreover, PRRT alone is relatively ineffective in patients with NET with a Ki67 proliferative index > 10%, a high tumor burden, and functioning tumors [9, 10, 17]. As a result, multidisciplinary treatment incorporating other therapies has been contemplated. In a retrospective analysis by Yordanova et al., the coadministration of an SSA with PRRT, or its use as a maintenance therapy, demonstrates significant improvement in PFS (48 months vs 27 months) and OS (91 months vs 47 months) when compared with PRRT alone [35]. Another multicenter study reported that the addition of lanreotide following PRRT elevated the objective response rate from 27.3% to 36.8% [36]. The combination of PRRT with other chemotherapies, as well as with SSAs, enhances the disease control rate [37, 38]. Initially, controlling unresectable liver and bone metastases with lanreotide therapy alone was challenging in our case. However, subsequent PRRT successfully suppressed tumor growth, and additional lanreotide therapy markedly reduced tumor size, consistent with the findings of previous studies [37, 38]. When reviewing cases of conversion surgery in PNETs with unresectable liver metastases, all previously reported patients received PRRT alone. However, in our patient, it was evident that additional SSA therapy radiologically suppressed disease progression and contributed to the histological disappearance of the tumor (pCR).

The present case involved metachronous liver metastasis after primary NET resection, indicating the application of drug therapy combined with PRRT. However, in cases of simultaneous unresectable liver metastases, primary NET resection may serve as a therapeutic option to enhance the efficacy of PRRT. Evidence indicates that reducing tumor volume through primary NET resection leads to a favorable response to PRRT of metastatic tumors, thus improving prognosis [17]. Once the disease is exclusively confined to the liver, subsequent PRRT combined with chemotherapy is expected to be effective.

The present case report has several limitations. First, it reports a single case. However, NETs are rare, and it is even more challenging to collect resection cases after PRRT. Previous studies of conversion surgery employed few patients [19–21, 31–33]. Although conducting a randomized controlled trial will be difficult, an increase in successful conversion surgery after PRRT may result in more opportunities to obtain a pCR in the future. With the accumulation of patients undergoing conversion surgery, it may be possible to establish resection criteria and optimal timing for conversion surgery after PRRT. Second, while our patient could safely complete PRRT, adverse events should always be taken into consideration. In phase III NETTER-1 study [12], 85% of patients experienced adverse events during PRRT with 177Lu-DOTATATE, including grade 3 or higher neutropenia, thrombocytopenia, and lymphopenia, and only 77% of patients completed the planned four cycles of PRRT. Myelodysplastic syndromes were also observed in 0.9% of patients. Therefore, prompt diagnosis and treatment of adverse events are essential during PRRT. Third, this patient is currently undergoing follow-up 17 months after conversion surgery. In general, PNET progresses slowly, and cases of progression from residual tumors occur four years postoperatively in the conversion case series [20]. Therefore, in our case, long-term follow-up is necessary to ensure the absence of recurrences or re-emergence of disappearing disease. Fourth, in our patient, we assessed the therapeutic effects of PRRT and SSAs through CT/MRI and confirmed that liver and bone metastases were both well controlled before conversion surgery. Although tumor viability was not assessed by SRS before surgery, postoperative follow-up SRS showed no evidence of metastases except that the bone metastasis showed mild accumulation of octreotide without apparent progression. We believe that this finding suggests conversion surgery was adequately performed. The application of ^111^In-pentetreotide and ^68^Ga-DOTATOC-PET as well as conventional CT, MRI, and SRS for detecting distant metastases and assessing the efficacy of preoperative treatment will be particularly important in more complex treatment protocols.

## Conclusion

We report a rare case of conversion surgery for initially unresectable multiple liver metastases after PNET resection. PRRT and SSAs significantly reduced the tumor burden, and a pCR was detected in resected liver specimens. Our case indicates the promising potential of PRRT and SSA therapy as a multimodal treatment option for unresectable liver metastases of PNET.

### Supplementary Information

**Additional file 1: Fig. S1.** A schema shows the treatment course and the tumor location.

## Data Availability

All data generated or analyzed during this study are included in this published article.

## Abbreviations

PNET: Pancreatic neuroendocrine tumor
PRRT: Peptide receptor radionuclide therapy
SSTR: Somatostatin receptor
SSA: Somatostatin analog
OS: Overall survival
PFS: Progression-free survival
CECT: Contrast-enhanced computed tomography
PPPD: Pylorus-preserving pancreaticoduodenectomy
MRI: Magnetic resonance imaging
SRS: Somatostatin receptor scintigraphy
SPECT: Single photon emission computed tomography
CR: Complete response
pCR: Pathological complete response
